# Effect of Monoclonal Antibody Blockade of Long Fragment Neurotensin on Weight Loss, Behavior, and Metabolic Traits After High-Fat Diet Induced Obesity

**DOI:** 10.3389/fendo.2021.739287

**Published:** 2021-10-08

**Authors:** Zherui Wu, Nicolas Stadler, Amazigh Abbaci, Jin Liu, Agnès Boullier, Nicolas Marie, Olivier Biondi, Marthe Moldes, Romain Morichon, Bruno Feve, Olle Melander, Patricia Forgez

**Affiliations:** ^1^ Inserm UMRS 1124 T3S, Paris University, Paris, France; ^2^ Department of Immunology, School of Medicine, Shenzhen University, Shenzhen, China; ^3^ MP3CV-UR7517, CURS-Université de Picardie Jules Verne & Laboratoire de Biochimie CHU Amiens-Picardie, Amiens, France; ^4^ CNRS, ERL 3649, Pharmacologie et thérapies des addictions, Paris, France; ^5^ Sorbonne University, INSERM UMRS 938, Centre de Recherche Saint-Antoine, Paris, France; ^6^ Institute of CardioMetabolism and Nutrition, Paris, France; ^7^ Sorbonne University, CRSA Cytométrie Imagerie Saint-Antoine, Paris, France; ^8^ AP-HP, Hôpital Saint-Antoine, Service Endocrinologie, CRMR PRISIS, Paris, France; ^9^ Department of Clinical Sciences Malmö, Lund University, Malmö, Sweden; ^10^ Department of Emergency and Internal Medicine, Skåne University Hospital, Malmö, Sweden

**Keywords:** LF NTS targeted therapy, neurotensin, obesity, behavior, metabolism

## Abstract

**Background:**

Obesity is a major public health problem of our time as a risk factor for cardiometabolic disease and the available pharmacological tools needed to tackle the obesity pandemic are insufficient. Neurotensin (NTS) is a 13 amino acid peptide, which is derived from a larger precursor hormone called proneurotensin or Long Form NTS (LF NTS). NTS modulates neuro-transmitter release in the central system nervous, and facilitates intestinal fat absorption in the gastrointestinal tract. Mice lacking LF NTS are protected from high fat diet (HFD) induced obesity, hepatic steatosis and glucose intolerance. In humans, increased levels of LF NTS strongly and independently predict the development of obesity, diabetes mellitus, cardiovascular disease and mortality. With the perspective to develop therapeutic tools to neutralize LF NTS, we developed a monoclonal antibody, specifically inhibiting the function of the LF NTS (LF NTS mAb). This antibody was tested for the effects on body weight, metabolic parameters and behavior in mice made obese by high-fat diet.

**Methods:**

C57bl/6j mice were subjected to high-fat diet (HFD) until they reached an obesity state, then food was switched to chow. Mice were treated with either PBS (control therapy) or LF NTS mAb at the dose of 5 mg/kg once a week (i.v.). Mice weight, plasma biochemical analysis, fat and muscle size and distribution and behavioral tests were performed during the losing weight period and the stabilization period.

**Results:**

Obese mice treated with the LF NTS mAb lost weight significantly faster than the control treated group. LF NTS mAb treatment also resulted in smaller fat depots, increased fecal cholesterol excretion, reduced liver fat and larger muscle fiber size. Moreover, mice on active therapy were also less stressed, more curious and more active, providing a possible explanation to their weight loss.

**Conclusion:**

Our results demonstrate that in mice subjected to HFD-induced obesity, a blockade of LF NTS with a monoclonal antibody results in reduced body weight, adipocyte volume and increased muscle fiber size, possibly explained by beneficial effects on behavior. The underlying mechanisms as well as any future role of LF NTS mAb as an anti-obesity agent warrants further studies.

## Introduction

Obesity has become a major public health problem in the developed world and increasingly so also in the third world’s population (1). Many factors from genetic to behavioral and food consumption habits have been invoked as the cause of the increase in obesity. Nevertheless, once the disease is established, it is difficult to overturn the accumulated weight. Outside of bariatric surgery, which remains the most effective treatment option for obesity and metabolic diseases (2), the loss of weight remains challenging for patients. Progress in medication to improve anti-obesity therapy- on top of dietary caloric restriction- needs to be made, and in this vein, we addressed the possible action of peripheral blockade of the long form neurotensin (LF NTS).

Neurotensin (NTS) is a 13-amino acid peptide originally identified in the hypothalamus (3). NTS was subsequently found to be produced by enteroendocrine cells of the small intestine, and released to the circulation to act locally or as a hormone (4–7). Three receptors which are stimulated by the NTS sequence are known, i.e. NTSR1, NTSR2 and NTSR3 (the latter also known as sortilin-1) (8). NTS and NTSR1 were also found in adrenal and myenteric plexus of the gastro-intestinal tract (9–12).

In the central nervous system, NTS acts as a neurotransmitter. NTS has been shown to modulate dopaminergic signaling and thereby have implications for psychotic diseases (13). Moreover, in the lateral hypothalamic area, NTS has been shown to be essential for mediation of leptin and ghrelin induced suppression of hunger and food intake (14, 15). On the other hand, in the small intestine where NTS secretion is stimulated by e.g. fat intake, studies comparing normal mice with mice genetically deficient for NTS found that NTS contributes to intestinal absorption of fat as well as high fat diet (HFD) induced obesity, hepatic steatosis and glucose intolerance without effects on food intake (16). It is not clear if and how the central and peripheral NTS effects interact, but stress has been shown to induce hypothalamic NTS secretion (17) and NTS is also expressed in the enteric nervous system (18) suggesting a possible interplay.

The NTS (13 AA) mature and biologically active peptide is produced after proteolytic cleavage of the NTS precursor of 170 AA sequence. A long fragment NTS (LF NTS), also referred to as proneurotensin, of 163 AA is also produced. LF NTS includes the NTS C-terminal sequence that binds to NTSR1, and exhibits the same biological activity as the mature peptide but with a higher stability (19). The NTS mature peptide is released in the blood stream after meals and fat intake and is quickly broken down and eliminated by the liver through the portal vein (20). Due to its very high lability, NTS peptide acts mostly at the vicinity of its release site (21). On the other hand, LF NTS is a considerably more stable polypeptide, and circulates long enough to reach a steady state in the human plasma (22). An increased plasma level of LF NTS is strongly related to insulin resistance (16) and hepatic steatosis (23) in humans. Moreover, high levels of LF NTS strongly and independently predicts the development of obesity, diabetes mellitus, cardiovascular disease and mortality (16, 22, 24, 25). As the levels of LF NTS are clearly variable between individuals, we hypothesized that increased levels of circulating LF NTS polypeptide may reflect NTS gene activation followed by the default cleavage of its precursor. We developed a neutralizing antibody specific to LF NTS, which was previously shown to inhibit tumor progression and restore chemotherapy response demonstrating the biological action of LF NTS polypeptide in a pathological context (26). This antibody was selected for its ability to bind the LF NTS and to neutralize the morphological changes of CHO cells overexpressing NTSR1 (27). The antibody inhibits the tumor growth of experimental cancer tumors emanating from diverse origins, all related to the over expression of NTSR1. This effect is absent when NTSR1 expression was lowered by employing sh-RNA (see patent EP14305825.3) (26).

Obesity is a metabolic disease characterized by uncontrolled accumulation in fat body stores. Obesity ensues from disbalance between energy intake and output attributable to complex interactions between biological, behavioral, social and environmental factors. Once obesity is established it is difficult to lose and maintain a healthy weight. Losing weight is in the majority of cases associated with reduced caloric intake, voluntarily or not, as ensuing bariatric surgery. In order to match the clinical situation of pharmacological obesity treatment which frequently associates medication and caloric restriction (28), we designed our experimental procedure to evaluate the role of inhibition of the LF NTS during the phase of caloric restriction after-induced obesity by HFD. Circulating LF NTS is enhanced upon inflammation associated with HFD consumption (29). Therefore, we hypothesized that by controlling the level of LF NTS we may alter the weight losing process. To be noted, mice on normal diet treated with long LF NTS mAb are more active and heavier than the control mice (26). In addition, we confirmed that LF NTS mAb treatment during the induction of obesity phase did not induce a reduction of weight accumulation.

Given the strong relationship between LF NTS and cardiometabolic disease, we set out to test whether and how blockade of LF NTS in the circulation with a monoclonal antibody that binds to LF NTS (LF NTS-mAb) affects body weight, metabolic parameters, and behavior in mice made obese by high-fat diet.

## Materials and Methods

### Animals

**Figure 1:**
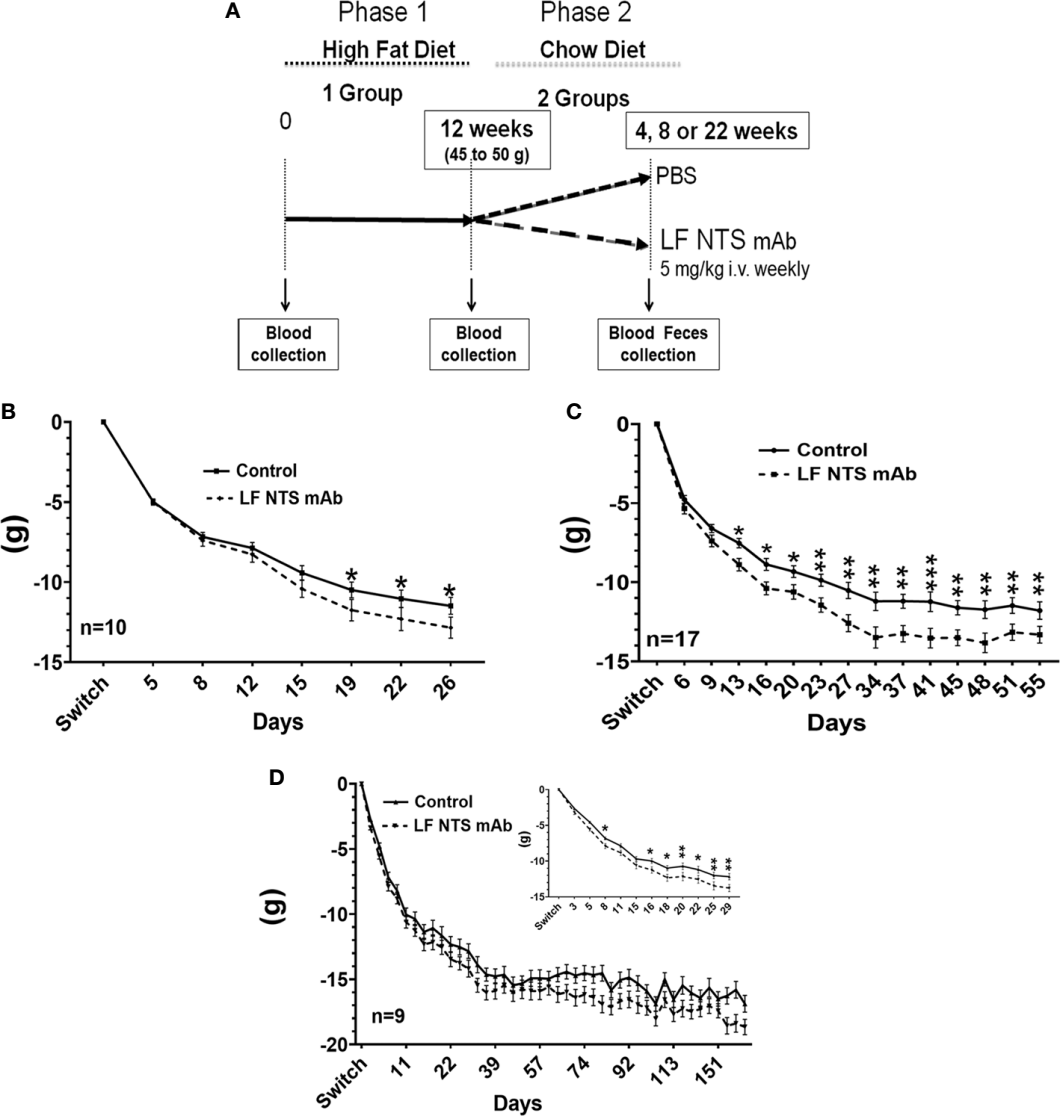
Weight lost by mice after the diet switch from high fat to chow diets. **(A)** Follow-up of the weight lost was performed on obese mice after the switch from HFD to chow and treated with FL-NTS mAb. Four independent experiments were performed. The weight lost was studied over different periods. **(B)** The first period while mice were losing weight was named a short period (SP) (n=10), **(C)** The second period corresponding to the weight stabilization was named long period (LP) (n=17) representing the mean of three independent experiments. **(D)** The LF NTS mAb was prolonged for 9 mice and maned long term (LT), (D inset).Magnification of the weight follow up during the first 29 days. Two-way ANOVA *p < 0.05; **p < 0.01; ***p < 0.001.

All experimental procedures were approved by the local ethical committee (APAFIS#9892). Mice were housed in a temperature (21 ± 1°C) and humidity (55 ± 10%) controlled room with a 12 h light, 12 h dark cycle (light on between 8 am and 8 pm). Food and water were available ad libitum. All experiments were performed with C57BL/6JRj male mice of six weeks old, considered as young adult. After a week of adaptation, mice were subjected to high-fat diet (HFD) for 12 weeks. The 260 HF HFD from Scientific Animal Food & Engineering was sugar and fat enriched diet with 60% of energy *via* butter. When mice reach between 45 to 50 g the food was switched to Chow diet, LASQCdiet^®^ Rod16-R form Lasvendi GmbH. Mice were randomly separated in two groups, treated in the retro-orbital sinus with either PBS (control therapy) or LF NTS mAb at the dose of 5 mg/kg once a week in a maximum volume of 100 µl (**Figure 1A**) (26). We previously used total mouse IgG as a control therapy, as it provides similar data to PBS for short-term treatments (26). Nevertheless, we find variable responses within mice and unexpected mouse deceases when treated for a long period, which led us to conclude that total IgG was not an inert control treatment. To be able to compare between experiments we used PBS as control therapy for all experiments. The results were collected from four separate experiments.

### Plasma Biochemical Analysis

Blood samples were collected from the retro-orbital sinus before the switch (HFD n=17), 26 (n=21) and 55 days (n=10) after the switch, and subsequently centrifuged at 1000 g for 10 min to obtain plasma for determination of the various clinical chemistry parameters. As control, 10 mice male treated with LF NTS mAb or PBS and maintained on chow diet were used. Assays were carried out using a benchtop biochemistry analyzer (RX Daytona+, Randox Laboratories Ltd, Roissy en France, France) according to the manufacturers’ protocol. After calibration of the instrument for each parameter, standard controls were run before each determination, and the values obtained for the different biochemical parameters were always within the expected ranges. Total cholesterol, triglycerides, HDL-cholesterol and glucose were quantified using enzymatically methods while total protein and albumin were photometrically determined.

### Fecal Lipid Content

Feces were collected from mice housed individually over a 48 h period at day 24 to 26 for SP (n= 11 for PBS group and 14 for LF NTS mAb group) and 48 to 50 for LP (n=8). Feces were dried at 60 C for 24 h, powderized in water (5 mL for 300mg) and then incubated with 5 mL of chloroform-methanol (2:1). After vortexing, the suspension was centrifuged at 1000 g for 10 min at room temperature. The lower liquid phase whose contains the extracted lipids was evaporated to dryness. Samples were then resuspended in 500 µL chloroform, 2% Triton X-100, evaporated to dryness and finally resuspended in 500 µL of water. Before analysis, samples were heated 10 min at 60°C. The final solvent concentration was then 2% Triton X-100 in water. Total cholesterol and triglycerides were then assayed using a benchtop biochemistry analyzer according to the manufacturers’ protocol (Randox Laboratories Ltd, Roissy en France, France). The calibrators and quality controls were diluted with 2% Triton X-100 in water.

### Lipid Extraction From Mouse Tissues

Tissues were collected at the time of the dissection and frozen SP (n=10) and LP (n=15). Tissues were weighted and homogenized with 1 mL chloroform/methanol (2/1) by the FastPrep-24. Lipids were extracted with a wash of the solvent with 0.9% NaCl solution on a rocker for 30 minutes at room temperature. The mixture was centrifuged at low speed (2000 g) to separate the two phases. The lower chloroform phase containing lipids was evaporated in the hood.

### OGTT and ITT

ITT (Insulin tolerance test) and OGTT (oral glucose tolerance test) were performed on a blood drop taken from the tail on mice fasted for 5 h. The drop was loaded on the strip and read with the OneTouch select plus reader (LifeScan Cilag GmbH international). OGTT was assayed after that animals were force-fed at 1 g/kg with a glucose solution. ITT was performed after i.p. injection of 1UI/kg insulin (Actrapid). OGTT was performed at day 12 (n = 7) and 51 (n = 7), ITT was performed at day 20 (n = 6) and day 58 (n = 7).

### Fat Size and Distribution

Adipose tissues were fixed in paraformaldehyde then paraffin embedded. Slides of 5 µm were stained with hematoxylin and eosin. Images were acquired with IX83 Olympus microscope and ORCA/4 Hamamatsu camera at objective 10 with phase contrast. Adipocyte sizes were obtained after binary transformation with ImageJ 1.53c software (30).

### Muscle Size and Distribution

Muscles were frizzed in OCT and kept at -80C. Immunocytochemistry was performed on slides of 4µm using Polyclonal Rabbit Anti-Laminin-1 (Dako) (1/1000) as primary antibody and Donkey anti Rabbit Alexa 555 (Invitrogen) as secondary antibody (1/1000). Images were acquired with IX83 Olympus microscope and ORCA/4 Hamamatsu camera at objective 10 with Mcherry filter. Muscle sizes were obtained after binary transformation with ImageJ 1.53c software (30).

### Behavioral Assessment

For these experiments, LF NTS mAb was injected i.v. at the dose of 5 mg/kg, once a week. The purified LF NTS polypeptide was injected at the dose of 1 µg/kg i.p. three times a week, alternatively in the right or the left side of the peritonea. PBS was used as control therapy. The LF NTS mAb and the LF NTS polypeptide experiments were performed independently. We noted that the baseline characteristics of the two control groups were different. This is explained by the different history of these two groups; the group treated with LF NTS mAb was 6 months old and exposed to HFD induced obesity followed by a fasting period, whereas the group treated with the polypeptide was 7 weeks old with no disturbance in diet.

#### Locomotor Activity

The total locomotor activity was measured in transparent activity boxes (19 cm x 11 cm x 14 cm) (Imetronic, France). Horizontal displacements and rearing activity were determined by photocell beams located across the long axis and above the floor. Locomotor activity was recorded during 48 hours, two active and one sleeping period with food and water ad libitum. Locomotor and rearing activity was expressed as the total number of interruption of the photocell beams. The locomotor activity was evaluated after the switch from HFD to chow over a period from day 22 to day 45 (n=16), and on mice treated with LF NTS for 3 to 4 weeks (n = 12).

#### Forced Swim Test

The forced swim test (31) was used to evaluate putative pro- or antidepressant-like effects of the LF NTS mAb, or of the purified LF NTS polypeptide. Mice were placed in a cylindrical jar filled with tap water at 23 ± 1°C to a sufficient depth (25 cm) to avoid the animal from touching the bottom or escaping the jar. The immobility time was measured for 4 min after a 2 min habituation period. The immobilization was defined as floating and moving limbs only to maintain the head over the water level. Four set of experiments were performed. In the first set, 5 mice were treated with LF NTS mAb (5mg/kg) or PBS during the high-fat diet induced obesity period. The forced swim test was performed when mice weight reached 49.9 ± 0.8 g and 47.18 ± 2g for control and treated mice, respectively. The second set was performed 50 days after the switch from HFD to chow (n=10), the third set 320 days after the switch (n = 9). For the fourth experiments, C57BL/6JRj male mice of five weeks old were treated with LF NTS for 4 weeks before performing the test.

#### Light/Dark Box Test

Effects of the LF NTS mAb or the purified polypeptide on anxiety were measured with the light/dark box test (32)). The apparatus used (43.5 cm x 26.5 cm x 26.5 cm) is composed of a black chamber (16.5 cm x 26.5 cm x 26.5 cm), and a white chamber (27 cm x 26.5 cm x 26.5 cm). The white compartment was illuminated at 650-700 lx, the black compartment at 25-35 lx. The chamber included an opening near the floor, to separate the two chambers. Animals were placed in the white compartment. We collected the time for the mouse to reach the dark chamber, the time spent in the dark chamber, and the number of transitions between the two compartments during a period of 5 min. Light/dark box test was performed at day 20 and 55 when mice were treated with LF NTS mAb (n=33). For the polypeptide fragment, LF NTS, 12 mice treated with for 2 to 4 weeks before performing the test.

### Statistical Analysis

All statistical analyses were performed using GraphPad Prism (GraphPad Software, Inc. La Jolla, USA). Continuous variables were compared between treatment groups using Two-way AN0VA or t-test, where appropriate. Dichotomous variables were compared between groups using chi-2 test.

## Results

### Weight Development During the Weight Lost Period

In order to evaluate the impact of the LF NTS mAb on body weight after high-fat diet induced obesity (HFD), we first fed the mice with HFD for 70 days until their weight reached 45-50 g, corresponding to a ~50% increase compared to their original weight. The food was switched to chow and mice were treated with LF NTS mAb or control therapy (PBS) (**Figure 1A**). **Figures 1B–D** refers to the average weight loss per group and shows that mice treated with LF NTS mAb lost more weight during three independent experiments with different durations of treatment. The experiments were ended either 20 to 30 days after the food switch i.e. short period (SP) (**Figures 1B, D** *inset*) or 45 to 55 days after the food switch, i.e. long period (LP) (**Figure 1C**), or continued long term, up to 170 days after the food switch) (LT) (**Figure 1D**).

In all experiments, the mice lost weight within the first 25 to 30 days, and then the weight stabilized for both groups (**Figures 1C, D**). **Table 1** shows the percentage of mice that lost at least 25% of their weight after food switch over time. In the group treated with LF NTS mAb, the percentage of mice reaching this goal increased with time (**Table 1**).

**Table 1:** Percentage of mice with 25% weight lost over time.

Day after switch	D13	D23	D37	D55
PBS	0%	24%	53%	59%
LF-NTS mAb	12%	53%	82%	94%
*Chi-square*	*P = 0.07*	*P = 0.04*	*P = 0.03*	*P = 0.009*

+Weight at switch 44.3 ± 1.1 g for PBS n = 17.

and 45.4 ± 0.8 g for LF-NTS mAb treated animals n = 17.

It is interesting to note that the difference in weight was maintained over time (LP) and exposure (LT) (**Figures 1C, D**). The food consumption was similar as well as the body temperature between the two groups during all experiments (data not shown).

### LF NTS mAb Limits the Cholesterol Absorption and Liver Steatosis

**Figure 2:**
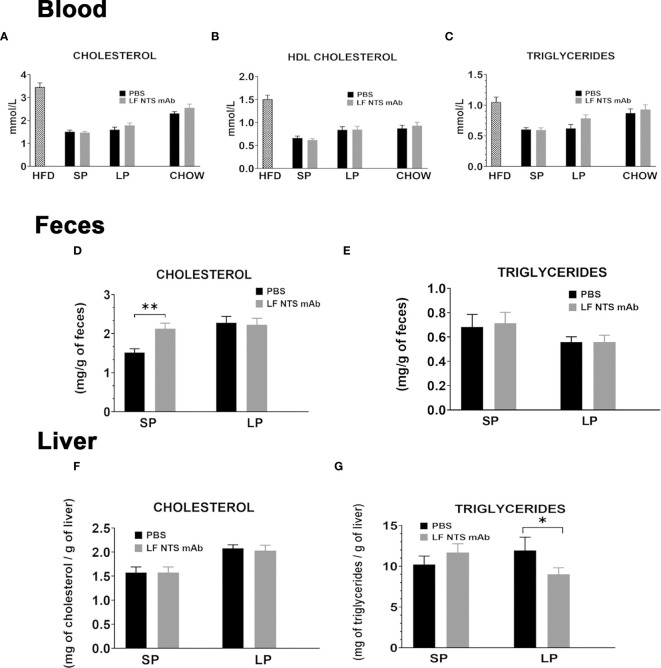
Lipid content evaluated at SP and LP. **(A–C)** Lipid contents were measured in plasma before the switch (n = 17) on to two set of mice; at SP mean on two independent experiments (n = 21) and at LP (n = 10). Lipid levels were compared to maintained in chow and treated or not with LF NTS mAb (n = 10). Lipid content in feces **(D, E)** collected during the two phases, and in the liver **(F, G)** liver at the dissection see details in the methods section. *t test* *p < 0.05; **p < 0.01.

Plasma lipid concentrations were evaluated in plasma during the SP and LP. Triglyceride, total cholesterol and HDL cholesterol levels were reduced after switch to chow regardless of therapy as compared to mice fed with HFD (**Figures 2A–C**). Plasma cholesterol and triglyceride levels were not modified by the LF NTS mAb, neither after SP nor after LP (**Figures 2A–C**).

Feces cholesterol content was significantly higher in animals treated with LF-NTS mAb during SP, then it stabilized and no difference between the two groups was detected during LP (**Figure 2D**). The triglyceride levels in the feces were not modified by the LF NTS mAb treatment (**Figure 2E**). The hepatic cholesterol content was equally not modified by the treatment (**Figure 2F**), whereas the triglyceride content decreased in animals treated with LF-NTS mAb during LP, suggesting LF NTS mAb facilitates elimination of the accumulated liver fat during the HFD, and therefore will reduce the liver steatosis (**Figure 2G**).

### Glucose Metabolism

**Figure 3:**
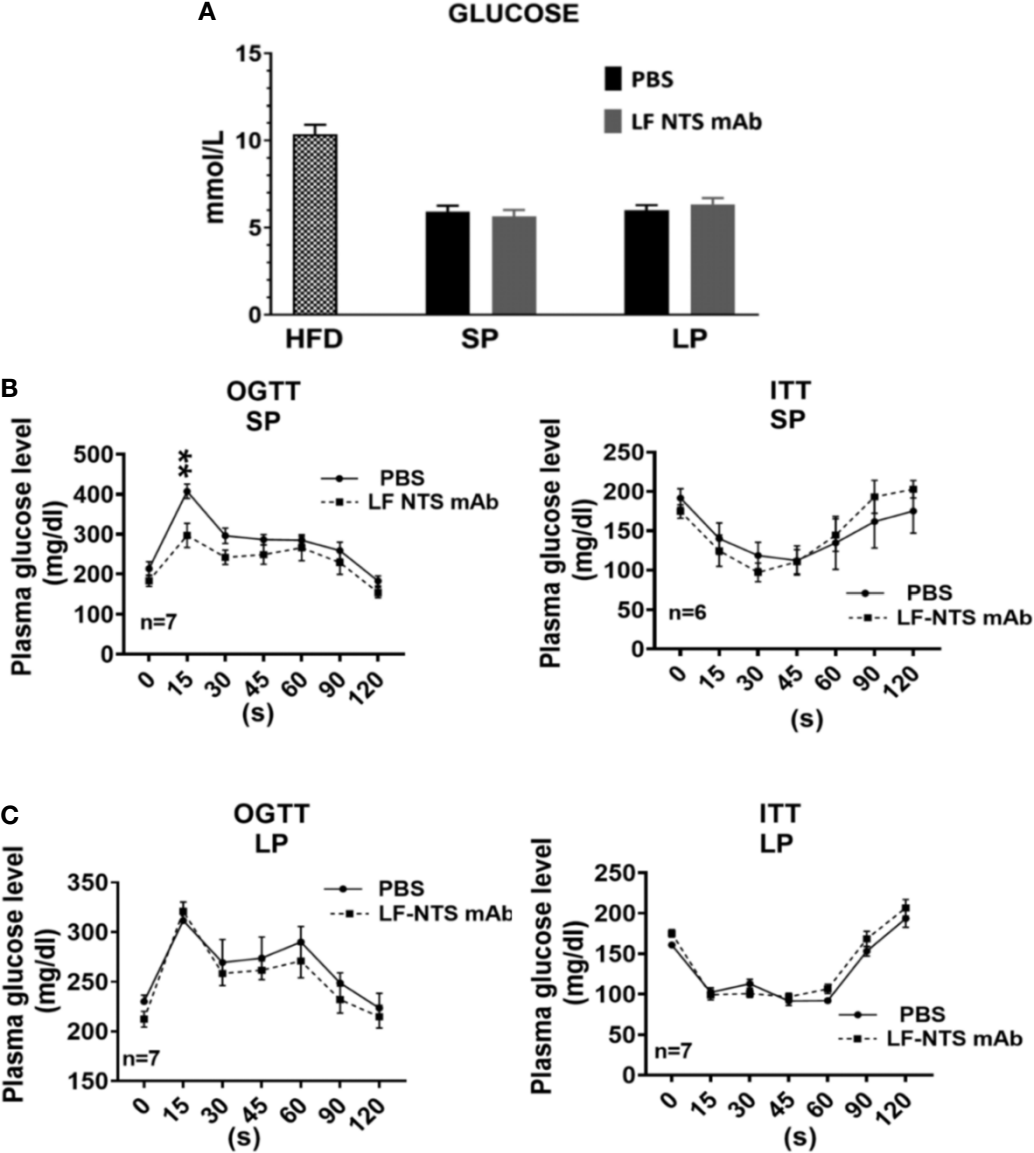
Glucose metabolism. **(A)** Glucose level were measured in plasma (HFD n = 17, SP n = 21, LP n = 10). Insulin tolerance test (ITT) and oral glucose tolerance test (OGTT) were performed during the SP **(B)** and LP **(C)** at 8 days apart. See details in the method section *2 way ANOVA* **p < 0.01.

As shown in **Figure 3A** the glucose level dropped massively after the switch from HFD to chow. During the weight losing period (SP and LP respectively), no differences were detected between the mice treated with LF NTS mAb and controls. OGTT and ITT tests were performed at SP (**Figure 3B**) and LP (**Figure 3C**), and no major differences were observed. Nevertheless, OGTT performed at SP suggested that the animals treated with LF NTS mAb experienced a better glucose tolerance than the control group, as the area under the curve of glucose was significantly different between the two groups p = 0.0393.

### Fat Size and Distribution

**Figure 4:**
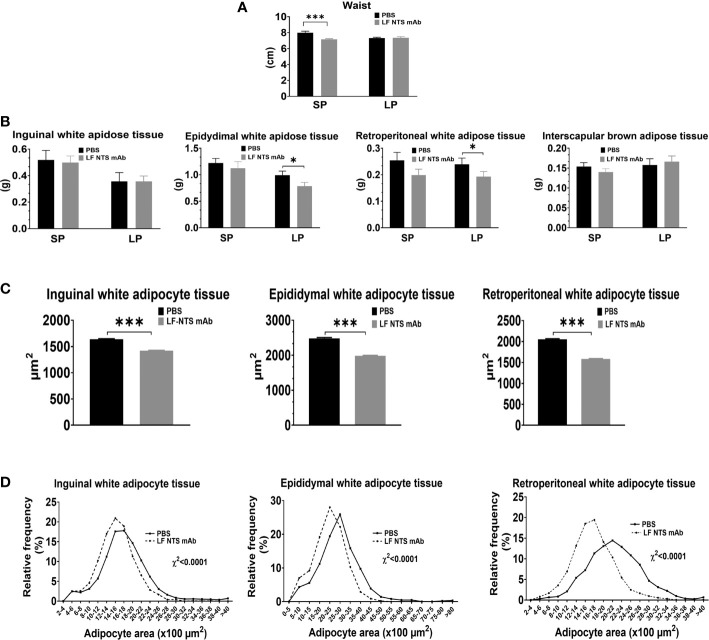
Adipocyte tissue analysis. **(A)** Waist size of dead mice at the dissection (n = 10), Adipose tissues was collected and weight immediately at the dissection. **(B)** weight of inguinal (n = 10), epididymal (SP n = 10 LP n = 18), retroperitoneal white adipose tissues (n = 10), and interscapular brown adipose tissue (SP n = 10 LP n = 18). Size of inguinal epididymal and retroperitoneal adipocytes was evaluated on mice treated by LF NTS mAb for SP (n = 10). **(C)** Mean size of the adipocytes and **(D)** adipocyte size distribution. *t test* *p < 0.05; ***p < 0.001.

During the SP of chow diet, the mice treated with LF NTS mAb appeared thinner and we confirmed this visual observation by measuring the abdominal circumference (waist) on dead mice assessed on the largest zone of the abdomen (**Figure 4A**). This difference was attenuated and disappeared with time (LP). The weight of the dissected adipose tissues in the epididymal and retroperitoneal compartments was significantly lower in mice treated with the LF NTS mAb only during the LP (**Figure 4B**), and no differences in weight were observed in the inguinal or brown fat interscapular compartments. Nevertheless, the analysis of the size and the size distribution of the adipocytes during SP showed that the adipocytes were emptied faster in mice treated with LF NTS mAb (**Figures 4C, D**). This is in agreement with the fastest loss of weight in LF NTS mAb treated mice.

### Muscle Size and Distribution

**Figure 5:**
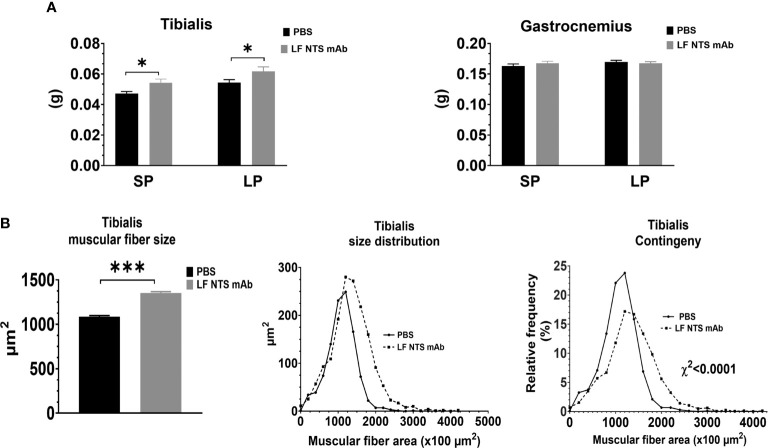
Muscle analysis. Muscles were collected and weight immediately at the dissection. **(A)** Weight of tibialis and gastrocnemius from 9 animals at SP and 16 at LP. **(B)** Size and size distribution of the tibialis muscular fibers at SP (n=8) *t test* *p < 0.05; ***p < 0.001.

At dissection times, the leg muscles were weighted. **Figure 5A** showed an increase in the weight of tibialis was observed all along the experiments (SP and LP), whereas no difference in weight was observed for the gastrocnemius muscle. Analysis of the muscular fiber size in the tibialis at SP confirmed larger fibers in mice treated with LF NTS mAb as compared to control mice (**Figure 5B**).

### Behavioral Tests

**Figure 6:**
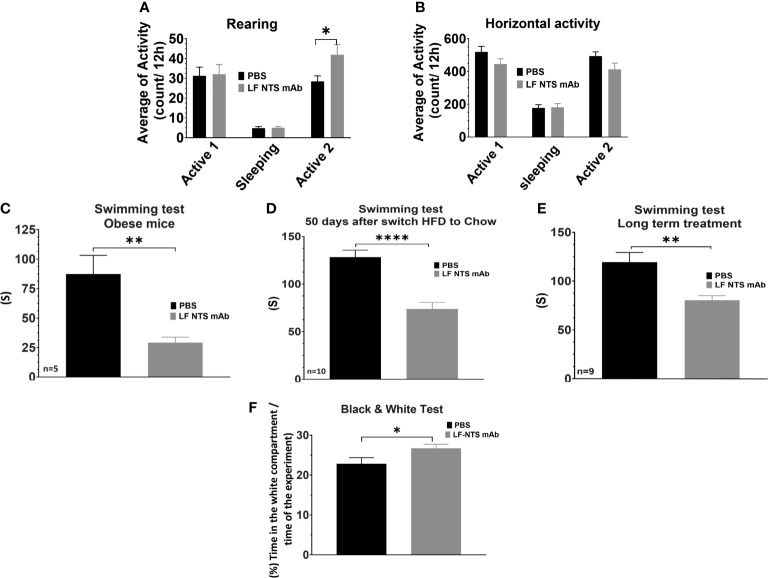
Behavioral tests upon FL-NTS mAb treatment. The locomotor activity tests were performed on mice treated with FL-NTS mAb after the switch from HFD to chow. **(A)** Rearing preformed over 48h, the calculation was performed during the two active phases (8pm to 8am) and a sleeping phase (8am to 8pm) **(B)** Horizontal activity. The graphs represent the average of 16 mice, performed from two independent experiments *t test* *p < 0.05. Swimming test performed on mice treated with FL-NTS mAb. **(C)** Test performed of mice feed with HFD for 12 weeks and treated of not with LF NTS mAb. The mice weighted 49.9 ±0.8 g and 47.18 ±2g for control and treated mice respectively (n = 5). **(D)** Test performed on obese mice and treated for 50 days after the switch to chow with the antibody or not (n = 10), **(E)** Test performed on obese mice and treated for 320 days after the switch to chow with NTS mAb or not (n = 9), see details in the method section. *t test* **p < 0.01; ****p < 0.0001. **(F)** Black and white test performed on mice treated with FL-NTS mAb after the switch HFD to chow. The graph represents the average of 33 mice performed on four independent experiments for the LF NTS mAb treatment. *t test* *p < 0.05.

Given the potential interplay between the peripheral and central nervous system NT systems and its potential impact on energy expenditure and thus body weight, we investigated whether LF NTS mAb treatment modified mice behavior. We first performed a locomotor activity test, which calculates the horizontal activity and the rearing. Rearing is an erect posture, adopted by the rodent with the intention of exploring. The **Figure 6A** revealed that mice treated with LF-NTS mAb seem to be more incline to explore after an adaptation period [second phase of activity]. No significant difference could be noticed in the horizontal activity **Figure 6B**. The force swimming test evaluated the depressive state of an animal. The **Figures 6C–E** clearly shows that mice treated with the LF NTS mAb were more inclined to fight to survive, suggesting a less depressive-like behavior.

The light/dark box test confirmed that the mice treated with the LF NTS mAb were less stressed than their littermates (**Figure 6F**). Mice have natural tendencies to avoid lighted and open areas. In this experiment, the time that animals spent in the white compartment was measured, indicating its enthusiasm and curiosity to explore novel environments. Animals spending more time in the white compartment will be less anxious. Here again, mice treated with the LF NTS mAb spent more time in the white compartment suggesting that treatment under LF NTS mAb reduces their fear.

**Figure 7:**
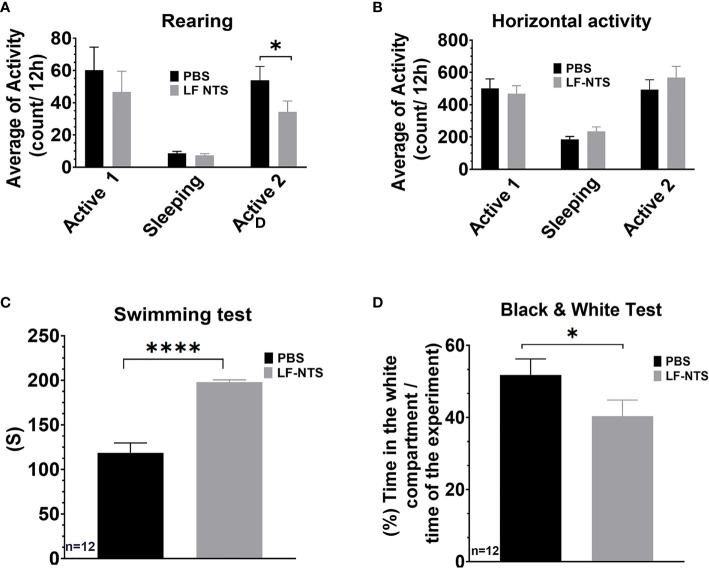
Behavioral tests upon FL-NTS polypeptide treatment. 12 Mice were treated with purified LF NTS three times a week with PBS or LF NTS polypeptide for 2 to 4 weeks. **(A)** Rearing preformed over 48h; the calculation was performed during the 2 active phases (8pm to 8am) and a sleeping phase (8am to 8pm). **(B)** Horizontal activity. **(C)** Swimming test. **(D)** Black and white test. t test. *p < 0.05; ****p < 0.0001.

These results indicate that the peripheral LF NTS can regulate the behavior of the animals. In order to apprehend this point, we performed the mirror experiment. Animals were treated with LF NTS polypeptides for two weeks before behavioral tests were performed. As compared with the effects of LF NTS mAb therapy, we now observed the reverse effects in all behavioral tests. Locomotor activity tests showed a reduced number of rearing events (**Figures 7A, B**), In swimming tests the mice gave up more easily, and in the black and white tests, mice spent the majority of time in the dark compartment (**Figure 7**). Together this data validates the possible role of LF NTS on animal stress and inhibition of stress by a neutralizing antibody. Food consumption and mouse weight were followed during a one-month period, and no differences could be observed between the PBS and the LF NTS treated mice [data not shown].

## Discussion

We demonstrate that in mice with HFD-induced obesity, chow diet in combination with LF NTS mAb therapy as compared to chow diet and control therapy resulted in significantly and greater weight loss. Importantly, even if differences in weight loss occurred during the first 25-30 days, the difference in weight remained over long term follow-up.

LF NTS mAb was originally developed under the hypothesis that during carcinogenesis and cancer progression, increased amount of active LF NTS is released from the transformed cells due to its overexpression and incomplete cleavage by specialized endoproteases. As the LF NTS is more stable than NTS in the circulation, we designed a targeted antibody to inhibit specifically LF NTS (26). Here, we demonstrated that LF NTS exerted a physiological function independently of the mature peptide. Although, LF NTS mAb was demonstrated to inhibit the NTSR1, treatment with LF NTS mAb on an integrated system as an animal might also directly or indirectly affect NTSR3 and NTSR2 stimulation and regulation. This point will need further studies and to be addressed independently to the different organs concerned.

In humans, high plasma concentration of LF NTS (proneurotensin), measured in the fasted state, predicts the development of obesity, metabolic syndrome, diabetes, and cardiovascular disease (22, 25, 33, 34). As the mature peptide is quickly broken down after meal stimulated release, the stable LF NTS remains in the circulation and it maintains receptor binding activity (19). We tested the hypothesis that the blockade of LF NTS in mice might reverse phenotypic features associated with high LF NTS in humans. Our results show that LF NTS mAb treatment in obese mice resulted in significantly greater weight loss and sustained lower weight.

Weight loss was accompanied by lower epididymal and retroperitoneal fat pads with smaller adipocytes suggesting that weight loss was driven by loss of fat. These findings represent the first piece of evidence suggesting that pharmacological blockade of LF NTS could serve as an anti-obesity therapy.

It has previously been demonstrated that knockout *NTS* mice are protected from development of HFD-induced obesity, liver steatosis, and glucose intolerance when compared to wild-type mice. This effect was, at least, partially driven by reduced intestinal lipid absorption and thus caused an increase of fecal excretion of lipids (16). Importantly, in the current study we wanted to mimic the clinical situation of pharmacological anti-obesity treatment as far as possible. As pharmacological anti-obesity therapy is frequently combined with dietary caloric restriction, we first induced obesity by HFD in the mice and then tested the LF NTS mAb vs control therapy after switching to chow (i.e. corresponding to “diet therapy”). We have tested a very similar experimental strategy as described by Li et al. (16), i.e. applying LF NTS mAb before and during induction of obesity with HFD to see if weight gain was prevented. We found that instead of lowering the weight accumulation as described for the NTS KO, mice treated with LF NTS mAb were heavier (unpublished data). This finding was confirmed by the previous experiments showing that LF NTS mAb induced an increase of weight in mice maintained on chow diet (26), and by the prevention of cachexia induced by cancer by LF NTS mAb (patent PCT/EP2019/073991). Together these observations suggest that LF NTS acts independently of the mature peptide and preferentially in the periphery, since it is admitted that only 0.1% of injected mAb cross the blood-brain barrier (35). As LF NTS mAb was efficient under diverse physiological and pathological circumstances, suggests that LF NTS effects are independent of the obesity process *per se*. LF NTS seems to be associated with a fine-tuning of the body physiology.

Here we applied pharmacological blockade of LF NTS after obesity was induced. Several similar phenotypic consequences were detected, as in the knockout model, but also several differences were revealed. Importantly, like in the NTS knockout mice, we observed no effect of LF NTS mAb on food intake, so differences in weight loss must have other causes than altered appetite and food intake. Although liver triglyceride content was not altered after SP, it was significantly reduced after LP in mice treated with LF NTS mAb, which is concordant with the key phenotypic feature of mice lacking the NTS gene. Whether the reduction of liver fat by LF NTS mAb is caused by reduced intestinal lipid absorption is not clear, as fecal secretion of triglycerides was not affected by therapy, whereas, fecal cholesterol secretion was significantly increased in animal’s treated with LF NTS mAb. To be noted, a higher elimination of cholesterol by the feces was previously observed in mice treated with LF NTS mAb and maintained on chow diet (26). Moreover, LF NTS mAb did not induce any consistent effects on glucose tolerance, although at SP, an improvement was observed.

Interestingly, despite greater weight loss and sustained lower body weight, LF NTS mAb treated mice got larger tibialis muscle and muscle fiber size. This finding prompted us to examine the effect of therapy on mice activity and behavior. In these experiments, LF NTS mAb blockade resulted in significantly more exploring and less depressive and anxiety signs, whereas LF NTS stimulation resulted on the opposite behavioral consequences. In combination with increased tibialis muscle size, these behavioral changes strongly suggest that the observed weight loss, due to loss of fat, following LF NTS mAb treatment is caused by enhanced activity probably associated with a behavior in favor of increase of physical activities. It is important to note that these effects were observed independently of the physiological and physical state of the animals, maintained on normal diet (26), or after HFD induced obesity as described here.

A major finding was that the neutralization of peripheral LF NTS altered animal’s behavior. It was previously shown that central NTS stimulates the activity of the hypothalamic-pituitary CRH-adrenocorticotropin hormone (ACTH) system (36). More recently, NTS has been suggested as a neuropeptide involved in stress-induced anxiety-like behaviors in the rats (37). It is unlikely that LF NTS polypeptides cross the blood-brain barrier and reach the synaptic cleft involved in the neurotensinergic transmission. It is also reasonable to hypothesize that the antibody does not cross the blood brain barrier either (35). A possible alternative explanation of the observed impact of LF NTS and mAb LF NTS on the behavioral tests might be interference with peripheral neurocrine or hormonal neurotensinergic regulation. Our studies raise the arguments for developing further experiments focusing on the role of LF NTS on the peripheral nervous system compartment and the hormonal or neuronal connection with the central nervous system and the hypothalamic-pituitary-adrenal axis. Moreover, an indirect interplay with leptin signaling also warrants further study, although we observed no difference in food intake. More sophisticated behavioral tests will also be needed to complete the description of the LF NTS effect on the animal’s manners. Also, lack of determination of LF NTS levels in animals treated with LF NTS mAb and control treated animals constitutes a limitation of our study, which should be addressed in further studies.

The large variability of LF NTS levels between individuals, and its string and independent association with disease development in humans (22), raised the question of the patho-physiological context associated with LF NTS overproduction. Genetic variability is one hypothesis, but until now, genetic studies (e.g. genome wide association studies) have not demonstrated convincing evidence of major genetic influence of LF NTS. This leaves open the possibility of transcriptional regulation. The NTS gene is stimulated upon injury, and mainly related with inflammatory processes (HFD, infection, allergy, cancer, wound, etc.) (26, 29, 38–40). Especially, it was shown that HFD intake in rat is related to an increase in LF-NTS plasma levels and peripheral inflammatory makers (29, 41). Moreover, intriguingly, human weight loss after gastric bypass surgery has been shown to be accompanied by increased levels of LF NTS (42). This highlights the complexity of how the NTS system is regulated, challenges high level of LF NTS as “metabolically dangerous” and underlines the need of further studies of both long-term consequences of blockade of the system as well as possible rebound effects after therapy is ended.

Studies on the human NTS promoter showed multiple cis-regulatory elements including a proximal region containing a cAMP-responsive element (CRE)/AP-1-like element that binds both the AP-1 and CRE-binding protein (6, 43). These transcription factors are activated from regulatory pathway very sensitive to stress and inflammation. Indeed, CREB can be activated followed the stimulation of adrenergic, prostaglandins, and histamine receptors. In the same way that AP1 transcription factors are activated following growth factor and interleukin receptor activation, NTS gene regulation could be a target of the regulatory pathway when diverse stimuli occur. The role of LF NTS on stress regulated from periphery can be supported by the data showing that NTS enhances the release of both CRH-ir and ACTH-ir in rat adrenal medulla (44). Hypothetically, LF NTS could activate adrenal cells or sympathetic nervous system NTS receptors and regulate the anxiety-like behaviors. The inhibition of these pathways by LF NTS mAb suggests a regulation of the neurotensinergic system from the periphery, which could be controlled by peripheral medication.

In conclusion, we provide here the first evidence that pharmacological blockade of LF NTS shows anti-obesity effects, which is encouraging since high LF NTS in human is a strong predictor of obesity and its sequels. Moreover, our data suggests that the underlying mechanisms may at least partly involve both effects on the reduction of anxiety, depressive-like state, and reduced disposition fat in central compartments.

## Data Availability Statement

The raw data supporting the conclusions of this article will be made available by the authors, without undue reservation.

## Ethics Statement

The animal study was reviewed and approved by local ethical committee (APAFIS#9892).

## Author Contributions

ZW, NS, AA, and JL performed the experiments, analyzed the data and performed the relevant literature review. AB performed the lipid assays. NM conceived behavioral experiments. OB conceived the experiments in muscle. MM and BF provided important discussion and data analysis on metabolism. RM conceived computational analysis for adipocytes and muscles studies. OM and PF conceived and designed the study, and drafted the manuscript. All authors contributed to the article and approved the submitted version.

## Funding

This work was supported by the Swedish Heart and Lung Foundation (20180278), the Swedish Research Council (2018-02760), the European Research Council ERC-AdG-2019-885003 and the Novo Nordisk Foundation (NNF200C0063465), and Erganeo/Inserm Transfert grant MAT-PI-07563-A-09.

## Conflict of Interest

The authors declare that the research was conducted in the absence of any commercial or financial relationships that could be construed as a potential conflict of interest.

## Publisher’s Note

All claims expressed in this article are solely those of the authors and do not necessarily represent those of their affiliated organizations, or those of the publisher, the editors and the reviewers. Any product that may be evaluated in this article, or claim that may be made by its manufacturer, is not guaranteed or endorsed by the publisher.
